# Novel Improved Synthesis of HSP70 Inhibitor, Pifithrin-μ. In Vitro Synergy Quantification of Pifithrin-μ Combined with Pt Drugs in Prostate and Colorectal Cancer Cells

**DOI:** 10.3390/molecules21070949

**Published:** 2016-07-21

**Authors:** Aoife M. McKeon, Alan Egan, Jay Chandanshive, Helena McMahon, Darren M. Griffith

**Affiliations:** 1 Centre for Synthesis and Chemical Biology, Department of Pharmaceutical and Medicinal Chemistry, Royal College of Surgeons in Ireland, 123 St. Stephens Green, Dublin 2 D02 YN77, Ireland; aoifemckeon@rcsi.ie (A.M.M.); jaychandanshive@rcsi.ie (J.C.); 2 Shannon ABC, South Campus, IT Tralee, Clash, Tralee, Co., Kerry V92 CX88, Ireland; ae12@hw.ac.uk (A.E.); helena.mcmahon@staff.ittralee.ie (H.M.)

**Keywords:** synthesis, pifithrin-μ, heat shock protein 70, cancer, platinum, combination study, Chou-Talalay method

## Abstract

We describe a novel improved approach to the synthesis of the important and well-known heat shock protein 70 inhibitor (HSP70), pifithrin-μ, with corresponding and previously unreported characterisation. The first example of a combination study comprising HSP70 inhibitor pifithrin-μ and cisplatin or oxaliplatin is reported. We have determined, using the Chou-Talalay method, (i) moderate synergistic and synergistic effects in co-treating PC-3 prostate cancer cells with pifithrin-μ and cisplatin and (ii) significant synergistic effects including strong synergism in cotreating HT29 colorectal cancer cells with oxaliplatin and pifithrin-μ.

## 1. Introduction

**Scheme 1:**
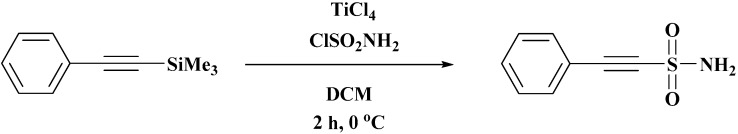
Previously reported synthetic strategy for synthesis of pifithrin-μ [6].

Pifithrin-μ, (2-phenylethynesulfonamide, PES, Scheme 1) is a potent and selective small molecule and direct inhibitor of heat shock protein 70 [1,2]. Heat shock proteins (HSPs) , extensively studied in the literature, are highly conserved proteins whose expression is increased by cells in response to a variety of cellular stresses including elevated temperatures, hypoxia, and anti-cancer chemotherapy for example [3]. There are at least eight highly homologous members of the human HSP70 family, which can be loosely organised by subcellular localisation, tissue-specific expression and stress induced expression.

HSP70-1 is a stress-inducible chaperone, which maintains protein homeostasis during normal cell growth but during a stress response is overexpressed and binds to and stabilises its protein substrates against denaturation or aggregation until adverse conditions improve [3]. It is an exciting anti-cancer target as it is overexpressed in colorectal and prostate cancers, amongst others, and is associated with cancer progression, chemotherapy resistance and poor prognosis. It is thought to provide cancer cells with a survival advantage by conferring protection against apoptosis, influencing senescence and inhibiting autophagy and HSP90 function [4].

Pifithrin-μ has been reported to interact with the C-terminal substrate (peptide) binding domain of HSP70 [1,5] and in doing so disrupts the association between HSP70 and (i) a number of its cofactors such as HSP40 and (ii) client proteins including APAF-1 (apoptotic peptidase activating factor 1, a key protein in the apoptosis regulatory network), p53 (a tumour suppressor protein) and p62 (autophagy-related proteins) [1]. Significantly pifithrin-μ selectively kills cancer cells via a caspase-independent mechanism involving increased protein aggregation, impairment of the autophagy-lysosomal system and the proteasome pathway, as well as indirectly effecting the activity of HSP90 [1,2].

To date only one synthesis of pifithrin-μ is reported in the literature, as Scheme 1; reaction of trimethyl(phenylethynyl)silane in dichloromethane (DCM) with a titanium sulfamoyl chloride complex, ClSO_2_NH_2_-TiCl_4_, in a reported 35% yield. No specific data associated with the characterisation of pifithrin-μ was provided [6].

**Figure 1:**
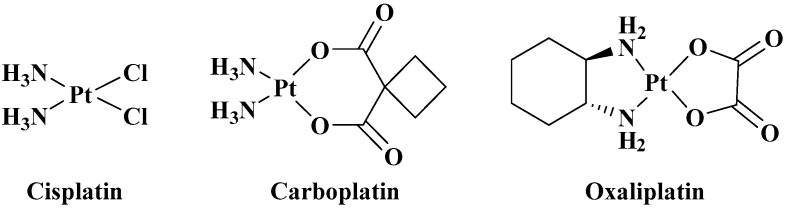
Structures of cisplatin, carboplatin and oxaliplatin.

Over the past 30 years, platinum (Pt) compounds have played a very important and well documented role in treating cancer and currently Pt-based drugs are employed in nearly 50% of anti-cancer therapies or regimens [7,8]. Pt drugs; cisplatin, carboplatin and oxaliplatin, Figure 1, are in worldwide clinical use, whereas nedaplatin, lobaplatin and heptaplatin are solely approved for use in Japan, China and South Korea, respectively. The cytotoxicity of Pt drugs is attributed to multiple mechanisms [9] but, primarily, their ability to enter cells, hydrolyse (loss of chlorido or carboxylato ligands) and covalently bind DNA, forming DNA adducts. These events can lead to DNA damage responses, senescence and ultimately programmed cell death, apoptosis [7,8,9].

Though Pt drugs are associated with high rates of clinical responses, many cancers including colorectal and prostate cancers, for example, are intrinsically resistant to Pt-based therapies. In addition, many cancers acquire chemoresistance, which further compounds therapeutic failure and tumour recurrence over the longer term [9].

Several mechanisms that account for the Pt resistant phenotype of tumour cells have been described in a recent review by Galluzzi et al., and they can be classified according to their position relative to Pt binding DNA, the accepted primary target; (i) pre-target resistance; (ii) on-target resistance; (iii) post-target resistance; and (iv) off-target resistance. Off-target resistance affects pathways that positively regulate pro-survival signals that nullify or diminish cisplatin cytotoxicity although they are typically not directly activated by cisplatin. The up-regulation of HSPs for example has been associated with off-target resistance [9]. 

Though metal complexes have a crucial role to play in anti-cancer treatment, it is apparent that most effective clinical regimens involve the use of combination therapy and that strategies to circumvent resistance should target at least two distinct mechanisms [7,9]. Consequently, we are interested in investigating if the HSP70 inhibitor pifithrin-μ can synergistically enhance the activity of Pt drugs. 

Herein, we describe (i) a novel improved synthesis of HSP70 inhibitor pifithrin-μ and (ii) an in vitro cytotoxicity combination study of a Pt drug and pifithrin-μ against prostate and colorectal cancer cells using the state-of-the-art method for determining synergy, the Chou-Talalay method.

## 2. Results and Discussion

### 2.1. Syntheses of Pifithrin-μ, PES

To date, only one synthesis of pifithrin-μ is reported in the literature, as Scheme 1; reaction of trimethyl(phenylethynyl)silane in DCM with a titanium sulfamoyl chloride complex, ClSO_2_NH_2_-TiCl_4_, in a reported 35% yield. No specific data associated with the characterisation of pifithrin-μ was provided [6].

**Scheme 2:**
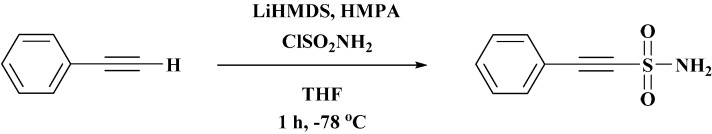
Novel A synthetic strategy for synthesis of pifithrin-μ.

We report a facile, reproducible and improved synthesis of pifithrin-μ Reaction of commercially available phenylacteylene with the organosilicon base LiHMDS (Li salt of hexamethyldisilazane) in anhydrous THF in the presence of HMPA (hexamethylphosphoramide), gives the corresponding acetylide anion, which subsequently reacts in situ with freshly prepared sulfamoyl chloride to give pifithrin-μ in 68% yield and excellent purity, Scheme 2. Pifithrin-μ was characterised by ^1^H- and ^13^C-NMR spectroscopy, mass spectrometry and elemental analysis. In the ^1^H-NMR spectrum of pifithrin-μ (DMSO-*d*_6_) three resonances, a doublet at 7.61 integrating for two, a triplet integrating for one at 7.56 and a triplet integrating for two at 7.48 ppm, correspond to the five protons of the aromatic ring. The resonance observed at 8.24 ppm is attributed to the two protons of the sulfonamide NH_2_. In The ^13^C-NMR spectrum signals at 132.2 (2 × C), 131.2 (1 × C), 129.2 (2 × C) and 117.9 (1 × C) ppm are associated with the six aromatic carbons and signals at 87.5 and 84.3 ppm are assigned to the two alkyne carbons. ESI-MS in the negative mode assisted in identifying pifithrin-μ with a mass peak at 180.2 a.m.u. Elemental analysis correlated with required analysis for pifithrin-μ.

### 2.2. In Vitro Cytotoxicity 

The in vitro anti-cancer chemotherapeutic potential of cisplatin and pifithrin-μ were determined against three prostate cancer cell lines; LNCaP (androgen sensitive) and PC-3, and DU145 (androgen insensitive) with a view to selecting one relatively Pt resistant cell line from each cancer type for the combination study described below. IC_50_ is defined as the concentration of compound that inhibits cell proliferation by 50% relative to the untreated cells. All three cell lines were found to have very similar sensitivity to treatment with both cisplatin and pifithrin-μ for 72 h treatment; cisplatin (4–6 μM) and pifithrin-μ (17–22 μM), Table 1. The values for cisplatin correspond with previously reported IC_50_ values in the literature [10].

The in vitro anti-cancer chemotherapeutic potential of oxaliplatin, the Pt drug of choice for the treatment of stage IV colorectal cancer, and pifithrin-μ were determined against three colorectal cancer cell lines; HT29, LoVo and HCT116. HCT116 and LoVo were found to be most sensitive to oxaliplatin treatment with IC_50_ value of 0.3 and 0.5 μM, respectively, relative to the HT29 cells (9 μM). The values for oxaliplatin correspond with previously reported IC_50_ values in the literature [11,12].

HT29 and HCT116 had somewhat similar sensitivity when treated with pifithrin-μ (40 and 26 μM respectively), which reasonably correspond with the IC_50_ values determined for the prostate cancer cell lines (17–22 μM), Table 1. It is noteworthy that the LoVo cells are particularly sensitive to treatment with pifithrin-μ with an IC_50_ of 5 μM. 

Significantly little or no data on pifithrin-μ against any prostate or colorectal cancer cell lines has been reported in the literature, however, pifithrin-μ has been shown to have IC_50_ values ranging from 2.5 to 12.7 µM against a number of leukemic cell lines [13].

### 2.3. In Vitro Combination Study

Unsubstantiated claims of synergy however are prevalent in the literature. Determination of a greater combined effect than each drug alone does not necessarily indicate synergism for example. Such an observation can be a result of an additive effect or even a slight antagonism. Synergy should not be confused with enhancement, potentiation, or augmentation [14]. 

An in vitro cytotoxicity combination study (*n* = 3) was therefore undertaken using the Chou-Talalay method, the state of the art method for determining a synergistic, additive or antagonistic effect on co-treatment of cells with two or more drugs [14]. This method for drug combination is based on the median-effect equation derived from the mass-action law principle, which is considered the unified theory for the Michaelis-Menten equation, Hill equation, Henderson-Hasselbalch equation and Scatchard equation. These equations provide the theoretical basis for the resulting combination index (CI) equation, a quantitative definition for synergism where CI < 1, antagonism where CI > 1 and additive effect where CI = 1. Based on these algorithms, Compusyn, a computer software developed for single drug and drug combinations, generates CI indexes [14,15]. 

PC-3 cells and HT29 cells were investigated as representative examples of prostate and colorectal cancer cells respectively given their relative resistance to Pt treatment in the in vitro cytotoxicity study described, Table 1.

**Figure 2:**
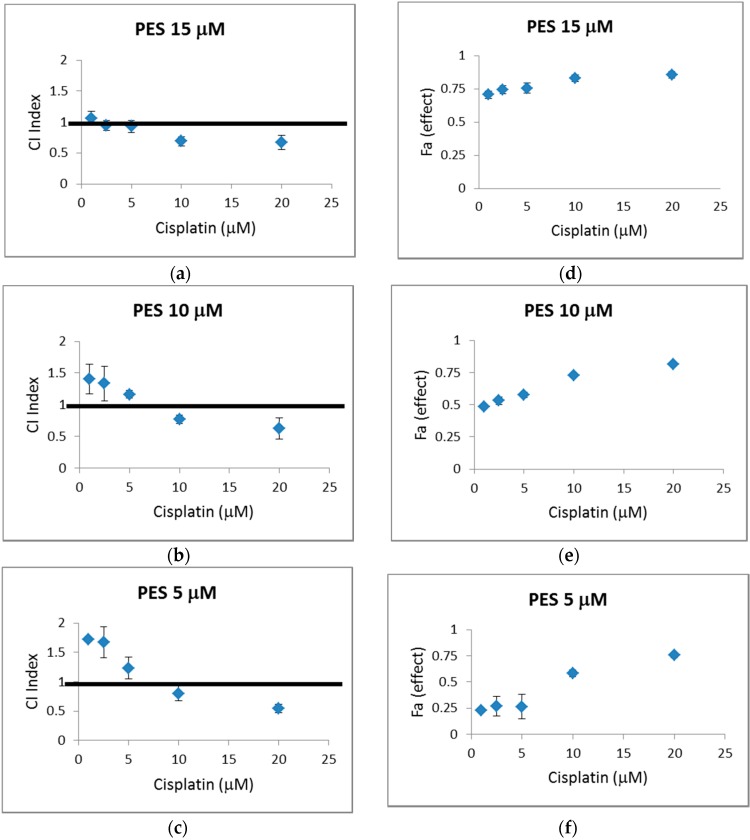
CI values for pifithrin-μ (PES) at 15 µM (**a**); 10 µM (**b**) and 5 µM (**c**) in combination with 1, 2.5, 5, 10, and 20 μM cisplatin against HT29 cells. CI (Combination index) is a quantitative definition for synergism where CI < 1, antagonism where CI > 1 and additive effect where CI = 1. The average ± SEM of three independent experiments relative to control, ≤0.2% DMSO treated cells. Fa effect for pifithrin-μ (PES) at 15 µM (**d**); 10 µM (**e**) and 5 µM (**f**) in combination with 1, 2.5, 5, 10, and 20 μM cisplatin. Fa (fraction affected) represents the respective proliferation inhibition, where 0% inhibition fa = 0% and 100% inhibition fa = 1. The average ± SEM of three independent experiments relative to control, ≤0.2% DMSO treated cells.

**Figure 3:**
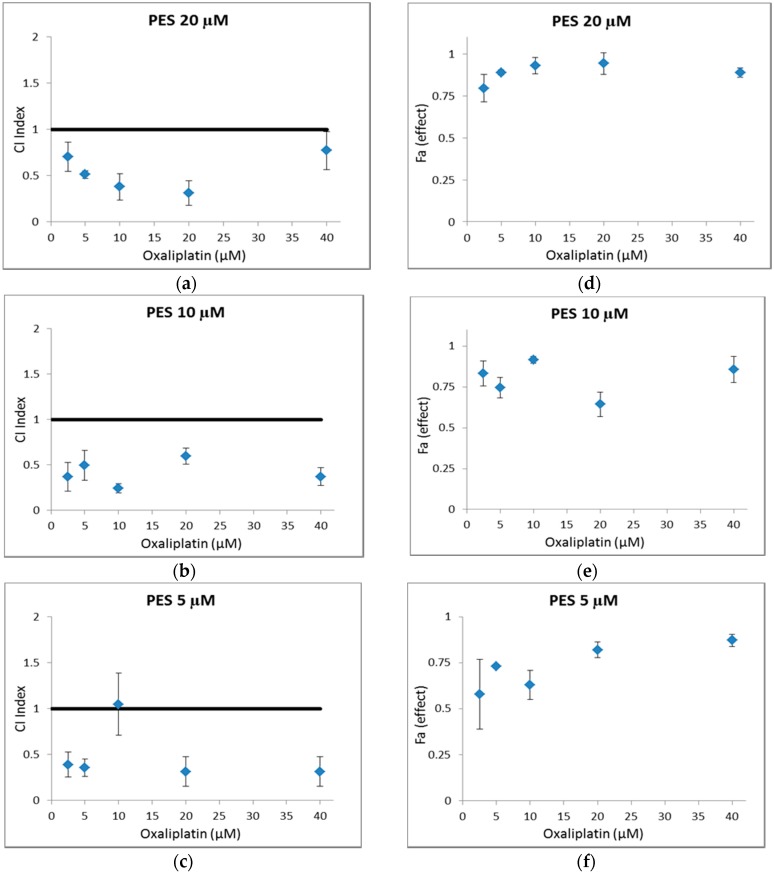
CI values for pifithrin-μ (PES) at 20 µM (**a**); 10 µM (**b**) and 5 µM (**c**) in combination with 2.5, 5, 10, 20 and 40 μM oxaliplatin against HT29 cells. CI (Combination index) is a quantitative definition for synergism where CI < 1, antagonism where CI > 1 and additive effect where CI = 1. The average ± SEM of three independent experiments relative to control, ≤0.2% DMSO treated cells. Fa effect for pifithrin-μ (PES) at 20 µM (**d**); 10 µM (**e**) and 5 µM (**f**) in combination with 2.5, 5, 10, 20 and 40 μM oxaliplatin. Fa (fraction affected) represents the respective proliferation inhibition, where 0% inhibition fa = 0% and 100% inhibition fa = 1. The average ± SEM of three independent experiments relative to control, ≤0.2% DMSO treated cells.

Both cell lines were treated with fixed ratios of pifithrin-μ and a Pt drug (cisplatin or oxaliplatin) as outlined in Table 2 and Table 3 where selection of concentrations was principally informed by the relevant IC_50_ values. Combination indexes were determined using Compusyn and plotted in Figure 2a–c and Figure 3a–c.

Of the 15 different combined concentrations of pifithrin-μ and cisplatin against PC-3 cells tested, seven combinations have CI values > 1 and eight have CI values < 1, Figure 2a–c. CIs can be interpreted in finer detail and categorised as Table 4 and as follows [15]. One combination, 10 μM pifithrin-μ and 5 μM cisplatin, with a CI of 1.16 exhibits slight antagonism, (CI category of 1.1–1.2), and three additional combinations exhibit moderate antagonism (CI category of 1.20–1.45). Three combinations (pifithrin-μ 15 μM and cisplatin 1, 2.5 and 5 μM) have CI indexes near to 1 and can be considered to exhibit a nearly additive effect (CI category of 0.9–1.1).

Two combinations, 5 μM pifithrin-μ and 10 μM cisplatin and 10 μM pifithrin-μ and 10 μM cisplatin fall in the moderate synergistic category (0.7–0.85) and four further combinations fall within the synergistic category (0.3–0.7). 5 μM pifithrin-μ and 20 μM cisplatin exhibit the lowest CI index of 0.55.

There are no previous reports in the literature of pifithrin-μ in combination with cisplatin against prostate cancer cells. Interestingly, the HSP70 inhibitor pifithrin-μ has been previously shown to increase the antitumor effects of hyperthermia against LNCaP, PC-3, and DU-145 prostate cancer cells [16].

Of the fifteen different combined concentrations of pifithrin-μ and oxaliplatin against HT29 cells tested, Figure 3a–c, one combination, 5 μM pifithrin-μ and 10 μM oxaliplatin, resulted in a CI value of c. 1, and is therefore considered to exhibit a nearly additive effect, Table 4.

The remaining fourteen combined concentrations tested resulted in the determination of CI values below 1 and in turn exhibit synergistic effects. 20 μM pifithrin-μ and 40 μM oxaliplatin resulted in a CI index of 0.77 which falls within the moderate synergism category of 0.7–0.85. In contrast 10 μM pifithrin-μ and 10 μM oxaliplatin with a CI value of 0.24 falls within the strong synergism category of 0.1–0.3. The remaining 12 combinations are found within 0.3–0.7 synergism category, Table 4. 

Given the importance of synergism being associated with high inhibition of cell proliferation fractions affected (fa, where 0% inhibition fa = 0% and 100% inhibition fa = 1) for all combinations investigated are plotted in Figure 2d–f and Figure 3d–f for ease of comparison with corresponding CI values. It is apparent that the majority of combinations associated with a synergistic value have a relatively high fa value.

Pifithrin-μ in combination with both oxaliplatin and cisplatin was determined to synergistically enhance the in vitro cytotoxic activity of the Pt drugs at particular concentrations and ratios and with corresponding high fa values. It is clear in this study that synergism was far more prevalent in the combination study undertaken for oxaliplatin and pifithrin-μ against the HT29 colorectal cancer cell lines as opposed to pifithrin-μ and cisplatin against the PC-3 cells. Similarly it is particularly noteworthy that the fa for the majority of combinations of pifithrin-μ and oxaliplatin against the HT29 cells were greater than 75%, Figure 3d–f.

The primary aim of identifying drug combinations is to achieve a synergistic therapeutic effect, dose and toxicity reduction, and to diminish or defer the onset of drug resistance [14]. Therefore HSP70 inhibitors, such as Pifithrin-μ, merit further investigation in combination with Pt drugs and alternative anticancer agents. 

## 3. Materials and Methods 

### 3.1. Materials and Instrumentation

Phenylacetylene, LiHMDS, HMPA, solvents and deuterated solvents were all purchased from Sigma Aldrich and used without further purification. Cisplatin [17], oxaliplatin [18] and sulfamoyl chloride [19] were synthesised via previously reported methods. IR spectra were recorded as KBr discs (400–4000 cm^−1^) on a Mattson Genesis II CSI FTIR spectrometer (Bruker, Billercia, MA, USA) and the spectra analysed using OPUS software (Version 5.0, Bruker). ^1^H- and ^13^C-NMR spectra were recorded on a Bruker Avance 400 NMR spectrometer and the spectra analysed using MestReNova software (version 6.0.2-5475, Mestrelab, Santiago de Compostela, Spain). The residual undeuterated DMSO signal at 2.505 ppm was used as an internal reference. Liquid chromatography-mass spectrometry experiments were performed on a Quattro Micro quadrupole electrospray mass spectrometer (Micromass, Waters Corp., Milford, MA, USA): 10 μL of the samples were injected in 300 μL of acetonitrile:water (60:40, *v*/*v*). The mass spectrometry data were acquired both in positive and negative ion modes. Analytical RP-HPLC was performed on a Perkin Elmer Series 200 apparatus (Perkin Elmer Inc., Waltham, MA, USA) using analytical column Intersil ODS-2 (150 Å, 5 µm); (A) Gradient: 0 to 40 min/0% to 100%; (B) flow rate = 1 mL/min^−1^; (C) UV detection was performed at 212 nm and (D) the solvent system consisted of 60% Acetonitrile/40% Water. Retention times (t_R_) from analytical RP-HPLC are reported in minutes. Elemental analysis (C, H, N) were performed by the RCSI Analytical Service, Department of Pharmaceutical and Medicinal Chemistry, Royal College of Surgeons in Ireland, 123 St. Stephen’s Green, Dublin 2, Ireland.

### 3.2. Syntheses of Pifithrin-μ

Phenylacetylene (0.54 mL, 4.9 mmol) was added to anhydrous THF (15 mL) and cooled to −78 °C using a mixture of liquid nitrogen and acetone. Once cooled, a 1 M solution of LiHMDS (5.4 mL, 5.4 mmol) was added dropwise and the mixture was left to stir for 10 min. HMPA (0.94 mL, 5.4 mmol) was then added before an additional 10 min of stirring. Freshly prepared sulfamoyl chloride (620 mg, 5.4 mmol) dissolved in anhydrous THF (5 mL) was subsequently added and the reaction mixture was left to stir for 1 h, maintaining the temperature at −78 °C. After stirring and returning to RT, EtOAc (15 mL) was added and the mixture washed with aqueous NH_4_Cl (15 mL). The aqueous layer was extracted with EtOAc (3 × 15 mL), the organic layers combined, dried over anhydrous Na_2_SO_4_ and concentrated under reduced pressure. The resulting crude oil was subject to column chromatography eluting with 30% EtOAc/hexane to yield pifithrin-μ as a white solid. Yield 0.603 g (68%). δ_H_ (400 MHz, DMSO-*d*_6_) 8.24 (2H, br s, NH_2_), 7.61 (2H, d, ^3^*J* 8 Hz, aromatic H), 7.56 (1H, t, ^3^*J* 8 Hz, aromatic H), 7.48 (2H, t, ^3^*J* 8 Hz, aromatic H). δ_C_ (100 MHz, DMSO-*d*_6_) 132.2 (aromatic C × 2), 131.2 (aromatic C × 1), 129.2 (aromatic C × 2), 117.9 (aromatic C × 1), 87.5 (alkyne C), 84.3 (alkyne C). (C_8_H_7_NO_2_S·½ H_2_O requires C, 50.52; H, 4.24; N, 7.36%. Found: C, 50.86; H, 4.67; N, 6.99%); HPLC: C18 column, isocratic 60% acetonitrile/40% water as an eluent, retention time: 4.19 min. Purity > 99%. MS (ESI-) *m*/*z*: 180.2.

### 3.3. In Vitro Cell Culture

Three prostate cancer cell lines; PC-3, LNCaP and DU145 were generously provided by Prof. Bill Watson, School Of Medicine, Conway Institute, University College Dublin and 3 colorectal cancer cell lines; HT29, LoVo, and HCT116 were kindly gifted by Prof. Jochen Prehn at the Department of Physiology and Medical Physics and the Centre for Systems Medicine at the Royal College of Surgeons in Ireland.

LNCaP cells were cultured in RPMI 1640 medium supplemented with 10% Heat-inactivated Foetal Bovine Serum (HI-FBS), PC-3 cells in Ham’s F12 Kaighn’s modified medium supplemented with 10% HI-FBS and DU145 cells in Modified Eagle Medium (MEM) supplemented with 10% HI-FBS.

HT29 and LoVo cell lines were cultured in DMEM (Dulbecco’s Modified Eagle Medium) supplemented with 1% penicillin-streptomycin, 1% l-glutamine and 10% FBS. The HCT-116 cells were cultured in RPMI 1640 supplemented with 1% penicillin-streptomycin, 1% l-glutamine and 10% FBS. Culture reagents and media were purchased from Biosera and used within 6 months of the purchase date.

In all cases cells were kept in an incubator set at 37 °C with 5% CO_2_. Together the CO_2_ environment and the hydrogen carbonate from the medium generate a physiology pH of 7.4. 

### 3.4. In Vitro Cytotoxicity Assays

One hundred microliters of stock solutions of cisplatin and oxaliplatin were made freshly in medium and diluted out with medium to required concentrations. A 20 mM DMSO pifithrin-μ stock solution was diluted out with medium to required concentrations with final DMSO concentration of ≤0.2%. 0.2% DMSO in medium was used as a control for experiments pertaining to pifithrin-μ. Cell growth was determined by the 3-(4,5-dimethylthiazol-2-yl)-5-(3-carboxymethoxyphenyl)-2-(4-sulfophenyl)-2*H*-tetrazolium, inner salt, (MTS test, Promega, Southampton, UK), a colorimetric assay based on the ability of viable cells to reduce a soluble yellow tetrazolium salt to blue formazan. 1 × 10^4^ prostate cancer cells or 3 × 10^4^ colorectal cancer cells were seeded per well onto 96-well plates in 100 µL of the appropriate culture medium. Twenty-four hours after seeding, the medium was removed and the cells were treated by adding 100 µL of the test compound solutions at appropriate concentrations. 

After 72 h of treatment, 20 µL of the MTS reagent was added to each well and the plates incubated for 2 hr at 37 °C. The absorbance was measured at 490 nm using a Wallac 1420 Victor 3 V plate reader (Perkin-Elmer Life Sciences, Boston, MA, USA). The percentage of surviving cells relative to untreated controls was then determined. The IC_50_ value defined as the drug concentration required to inhibit cell growth by 50%, was estimated graphically from dose-response plots using GraphPad Prisim, a scientific 2D graphing and statistics software (Prisim 5 for Windows, version 5.01, GraphPad Software Inc., La Jolla, CA, USA).

### 3.5. In Vitro Cytotoxicity Combination Study

A drug combination study was carried out and synergistic potential investigated using the Chou-Talalay method [14]. 

One hundred microliters of stock solutions of cisplatin and oxaliplatin were made freshly in medium and diluted out with medium to required concentrations. A 20 mM DMSO pifithrin-μ stock solution was diluted out with medium to required concentrations with final DMSO concentration of ≤0.2%. 0.2% DMSO in medium was used as a control for experiments pertaining to pifithrin-μ. Based on the in vitro cytotoxicity evaluation PC-3 prostate cancer cells and HT29 colorectal cancer cells were chosen for the combination study. PC-3 cells at a concentration 1 × 10^4^ or HT29 cells at a concentration of 3 × 10^4^ were seeded per well onto 96-well plates in 100 µL of the appropriate culture medium. Twenty-four hours after seeding the medium was removed and the cells were treated by adding 100 µL of the combinations in medium solutions at selected concentrations. Specifically, PC-3 cells were treated with combinations of pifithrin-μ and cisplatin, whereas HT29 cells were treated with combinations of pifithrin-μ and oxaliplatin, Table 2 and Table 3.

After 72 h of treatment, 20 µL of MTS reagent was added to each well and the plates incubated for 2 h at 37 °C. The absorbance was measured at 490 nm using a Wallac 1420 Victor 3V plate reader (Perkin-Elmer Life Sciences). The percentage of surviving cells relative to untreated controls were calculated and the fraction affected (fa) which represents the respective proliferation inhibition was determined, where 0% inhibition fa = 0% and 100% inhibition fa = 1.

The CI indexes were computed and graphically represented using dose-response plots in Compusyn [15].

## 4. Conclusions 

We described a novel improved synthesis for the important and well-known HSP70 inhibitor, pifithrin-μ, with corresponding and previously unreported characterisation. 

The first example of a combination study comprising HSP70 inhibitor pifithrin-μ and cisplatin or oxaliplatin is reported. We have determined moderate synergistic and synergistic effects in co-treating PC-3 prostate cancer cells with pifithrin-μ and cisplatin and significant synergistic effects including strong synergism in co-treating HT29 colorectal cancer cells with pifithrin-μ and oxaliplatin. 

This study indicates that HSP70 inhibition and Pt-based drug regimens should be investigated further as potential anticancer combination therapies. In addition the impact of co-treatment of pifithrin-μ and Pt drugs on cell death pathways merit investigation.

## Figures and Tables

**Table 1:** IC_50_ (μM) values calculated for cisplatin, oxaliplatin or pifithrin-μ against prostate and colorectal cell lines on 72 h treatment (*n* = 3).

Test Compounds	PC-3	DU145	LNCaP	HT29	LoVo	HCT116
Pifithrin-μ	17.4	18.8	21.6	40	5	26
Cisplatin	6.01	4.02	5.3	-	-	-
Oxaliplatin	-	-	-	9	0.5	0.3

**Table 2:** Combined drug concentrations investigated against PC-3 prostate cancer cells.

Cisplatin Concentration	Pifithrin-μ 15 µM	Pifithrin-μ 10 µM	Pifithrin-μ 5 µM
20 µM Cisplatin	20, 15	20, 10	20, 5
10 µM Cisplatin	10, 15	10, 10	10, 5
5 µM Cisplatin	5, 15	5, 10	5, 5
2.5 µM Cisplatin	2.5, 15	2.5, 10	2.5, 5
1 µM Cisplatin	1, 15	1, 10	1, 5

**Table 3:** Combined drug concentrations investigated against HT29 colorectal cancer cells.

Oxaliplatin Concentration	Pifithrin-μ 20 µM	Pifithrin-μ 10 µM	Pifithrin-μ 5 µM
40 µM Oxaliplatin	40, 20	40, 10	40, 5
20 µM Oxaliplatin	20, 20	20, 10	20, 5
10 µM Oxaliplatin	10, 20	10, 10	10, 5
5 µM Oxaliplatin	5, 20	5, 10	5, 5
2.5 µM Oxaliplatin	2.5, 20	2.5, 10	2.5, 5

**Table 4:** CI ranges and their descriptions using the Chou-Talalay method [14].

Combination Index (CI)	Description
<0.1	Very strong synergism
0.1–0.3	Strong synergism
0.3–0.7	Synergism
0.7–0.85	Moderate synergism
0.85–0.9	Slight synergism
0.9–1.10	Nearly additive
1.1–1.2	Slight antagonism
1.2–1.45	Moderate antagonism
1.45–3.3	Antagonism
3.3–10	Strong antagonism
>10	Very strong antagonism

## Supplementary Materials

Supplementary materials can be accessed at: http://www.mdpi.com/1420-3049/21/7/949/s1.
