# Correction: Griffith, D.M., et al. Novel Improved Synthesis of HSP70 Inhibitor, Pifithrin-μ. In Vitro Synergy Quantification of Pifithrin-μ Combined with Pt Drugs in Prostate and Colorectal Cancer Cells. *Molecules* 2016, *21*, 949

**DOI:** 10.3390/molecules21111550

**Published:** 2016-11-17

**Authors:** Aoife M. McKeon, Alan Egan, Jay Chandanshive, Helena McMahon, Darren M. Griffith

**Affiliations:** 1 Centre for Synthesis and Chemical Biology, Department of Pharmaceutical and Medicinal Chemistry, Royal College of Surgeons in Ireland, 123 St. Stephens Green, Dublin 2 D02 YN77, Ireland; aoifemckeon@rcsi.ie (A.M.M.); jaychandanshive@rcsi.ie (J.C.); 2 Shannon ABC, South Campus, IT Tralee, Clash, Tralee, Co., Kerry V92 CX88, Ireland; ae12@hw.ac.uk (A.E.); helena.mcmahon@staff.ittralee.ie (H.M.)

The authors are sorry to report that some of the ^1^H- and ^13^C-NMR data reported in their recently published paper [1] were incorrect. While this manuscript was in preparation the ^1^H- and ^13^C-NMR data and HPLC data for 2-chloro-2-phenylethene-1-sulfonamide were used as a placeholder. Consequently, the authors wish to make, at this time, the following corrections to the paper:

## 1. Change in Main Body Paragraphs

In the Section *2.1. Syntheses of Pifithrin-μ*, *PES*, this paragraph “In the ^1^H-NMR spectrum of pifithrin-μ (DMSO-*d*_6_) the two resonances, a multiplet at 7.47 integrating for three and a doublet integrating for two at 7.36 ppm, correspond to the five protons of the aromatic ring. The resonance observed at 7.29 ppm is attributed to the two protons of the sulfonamide NH_2_. In The ^13^C-NMR spectrum signals at 147.2 and 145.1 ppm are assigned to the two alkyne carbons and 139.5, 130.3, 128.7 and 128.5 ppm are associated with the six aromatic carbons. ESI-MS in the negative mode assisted in identifying pifithrin-μ with a mass peak at 180.2 a.m.u. Elemental analysis correlated with required analysis for pifithrin-μ.” was incorrectly reported.

It should be “In the ^1^H-NMR spectrum of pifithrin-μ (DMSO-*d*_6_), three resonances—a doublet at 7.61 integrating for two, a triplet integrating for one at 7.56 and a triplet integrating for two at 7.48 ppm—correspond to the five protons of the aromatic ring. The resonance observed at 8.24 ppm is attributed to the two protons of the sulfonamide NH_2_. In The ^13^C-NMR spectrum, signals at 132.2 (2 × C), 131.2 (1 × C), 129.2 (2 × C) and 117.9 (1 × C) ppm are associated with the six aromatic carbons and signals at 87.5 and 84.3 ppm are assigned to the two alkyne carbons.”

In the section *3.2. Syntheses of Pifithrin-μ*, this paragraph “δ_H_ (400 MHz, DMSO-*d*_6_) 7.44 (3H, m, aromatic H), 7.36 (2H, d, ^3^*J* 8 Hz, aromatic H), 7.33 (2H, br s, NH_2_). δ_C_ (100 MHz, DMSO-*d*_6_) 146.1 (alkyne C), 144.9 (alkyne C), 138.4 (aromatic C), 129.2 (aromatic C), 127.6 (aromatic C), 127.4 (aromatic C). (C_8_H_7_NO_2_S·½ H_2_O requires C, 50.52; H, 4.24; N, 7.36%. Found: C, 50.86; H, 4.67; N, 6.99%); HPLC: C18 column, isocratic 60% acetonitrile/40% water as an eluent, retention time: 8.63 min. Purity > 96%. MS (ESI-) *m/z*: 180.2.” was incorrectly reported.

It should be “δ_H_ (400 MHz, DMSO-*d*_6_) 8.24 (2H, br s, NH_2_), 7.61 (2H, d, ^3^*J* = 8 Hz, aromatic H), 7.56 (1H, t, ^3^*J* = 8 Hz, aromatic H), 7.48 (2H, t, ^3^*J* = 8 Hz, aromatic H). δ_C_ (100 MHz, DMSO-*d*_6_) 132.2 (aromatic C × 2), 131.2 (aromatic C × 1), 129.2 (aromatic C × 2), 117.9 (aromatic C × 1), 87.5 (alkyne C), 84.3 (alkyne C). (C_8_H_7_NO_2_S·½ H_2_O requires C, 50.52; H, 4.24; N, 7.36%. Found: C, 50.86; H, 4.67; N, 6.99%); HPLC: C18 column, isocratic 60% acetonitrile/40% water as an eluent, retention time: 4.19 min. Purity >99%. MS (ESI-) *m/z*: 180.2.”

## 2. Change in Figures in the Supplementary Material

The correct spectroscopic data (^1^H-NMR and ^13^C-NMR) and correct HPLC data (chromatogram and report) for pifithrin-μ are as follows (Figure S1, Figure S2, Figure S3, Figure S4, Figure S6 and Figure S7):

The authors would like to apologize for any inconvenience caused to the readers by these changes.

## Figures and Tables

**Figure S1:**
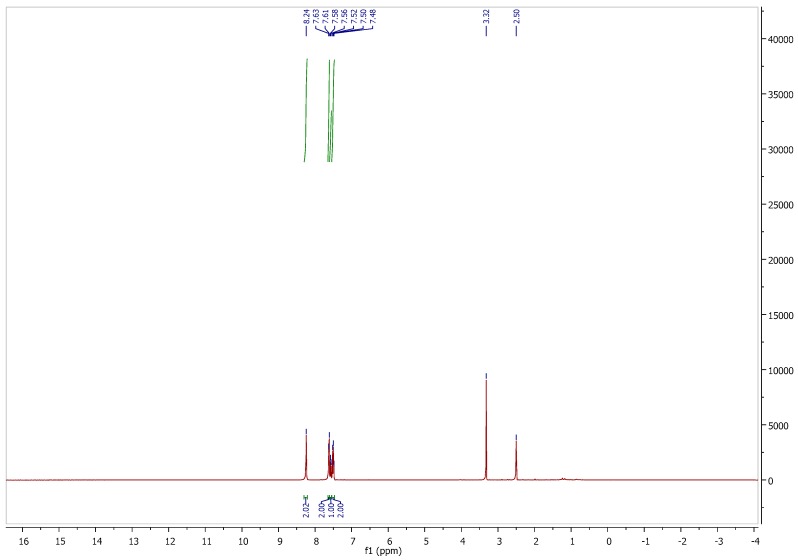
^1^H-NMR spectrum of pifithrin μ in DMSO-*d*_6_.

**Figure S2:**
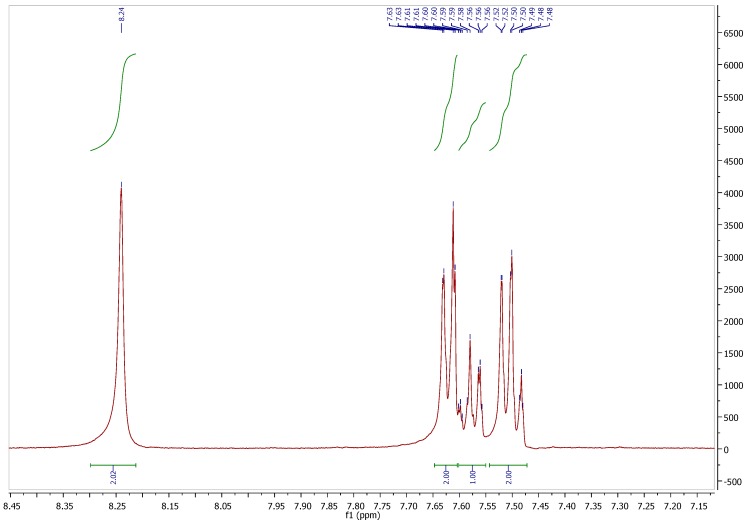
^1^H-NMR spectrum of pifithrin-μ in DMSO-*d*_6_.

**Figure S3:**
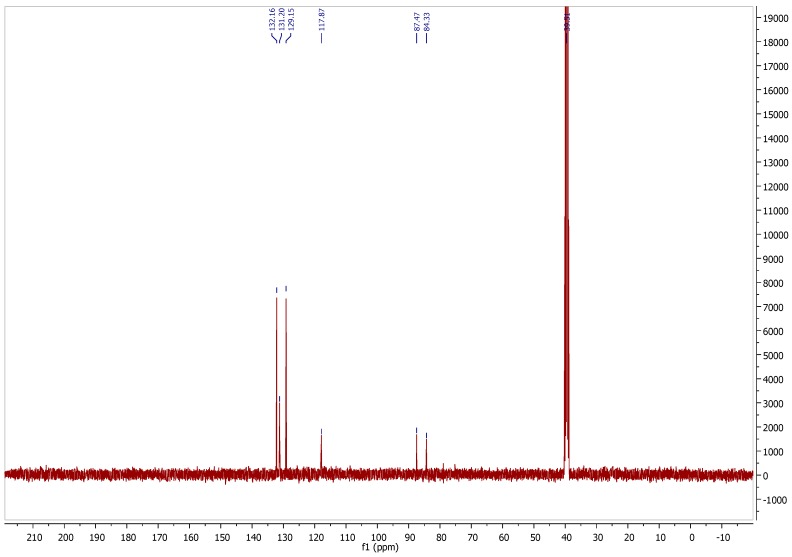
^13^C-NMR spectrum of pifithrin-μ in DMSO-*d*_6_.

**Figure S4:**
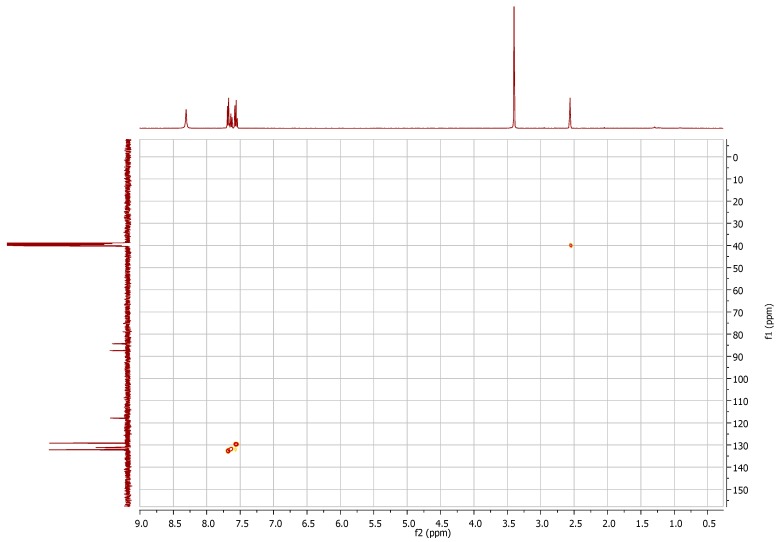
HSQC spectrum of pifithrin-μ in DMSO-*d*_6._

**Figure S6:**
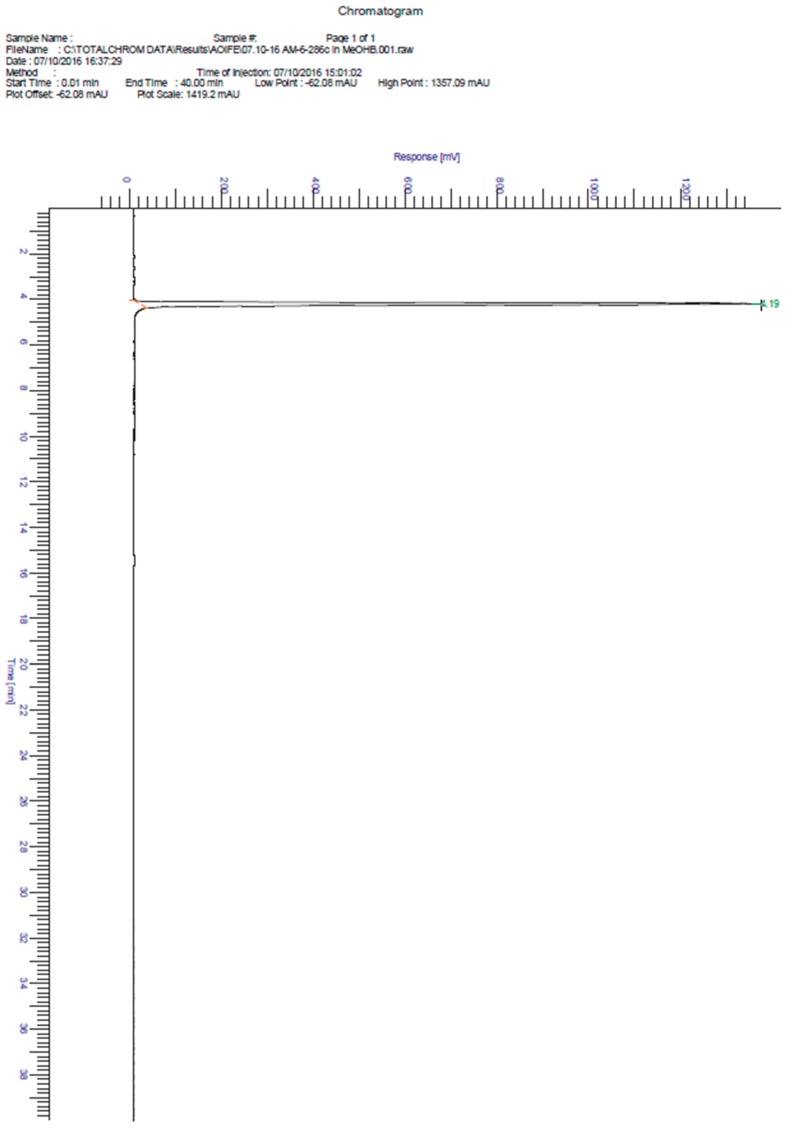
HPLC chromatogram of pifithrin-μ.

**Figure S7:**
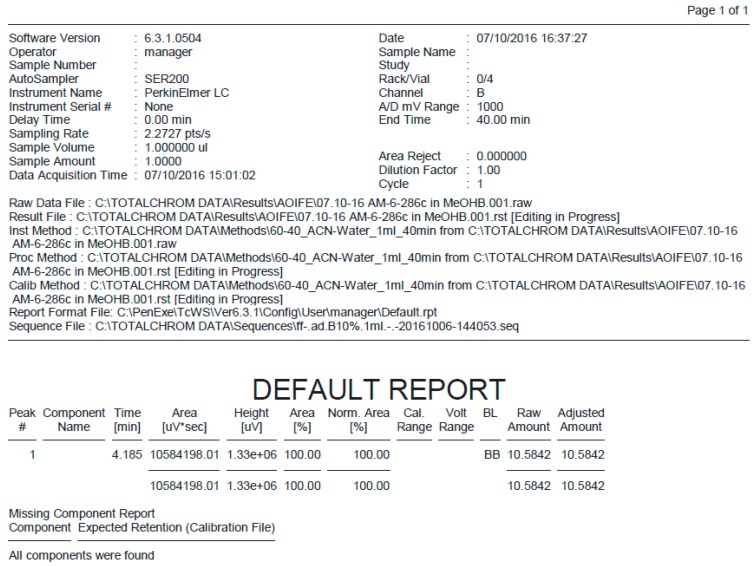
HPLC report for pifithrin-μ.
