# Supplementary material for: Novel Improved Synthesis of HSP70 Inhibitor, Pifithrin-μ. In Vitro Synergy Quantification of Pifithrin-μ Combined with Pt Drugs in Prostate and Colorectal Cancer Cells
Source: Molecules. 2016 Jul 21;21(7):949. doi: 10.3390/molecules21070949 (PMC6273252; doi:10.3390/molecules21070949)
Supplement: Supplementary file 1 [file molecules-21-00949-s001.pdf]

Supplementary Materials: Novel Improved Synthesis of HSP70 Inhibitor, Pifithrin μ. in vitro Synergy Quantification of Pifithrin μ Combined with Pt Drugs in Prostate and Colorectal Cancer Cells

Aoife M. Mckeon, Alan Egan, Jay Chandanshive, Helena McMahon, Darren M. Griffith

Table S1. Table representing the CI index and Fa calculated for oxaliplatin and pifithrin-μ at varying combined concentrations against HT29 colorectal cancer cells.

Oxaliplatin (μM)	PES (μM)	CI (*n* = 3)	SEM	Fa (*n* = 3)	SEM
40	20	0.771	0.205	0.888	0.027
40	10	0.372	0.097	0.857	0.079
40	5	0.315	0.161	0.872	0.033
20	20	0.312	0.135	0.942	0.063
20	10	0.595	0.089	0.643	0.075
20	5	0.315	0.161	0.821	0.044
10	20	0.381	0.142	0.931	0.049
10	10	0.244	0.050	0.916	0.022
10	5	1.048	0.339	0.631	0.079
5	20	0.512	0.040	0.890	0.015
5	10	0.496	0.166	0.745	0.062
5	5	0.355	0.095	0.732	0.006
2.5	20	0.704	0.157	0.795	0.082
2.5	10	0.372	0.158	0.833	0.076
2.5	5	0.390	0.135	0.579	0.190

Table S2. Table representing the CI index and Fa calculated for cisplatin and pifithrin-μ at varying combined concentrations against PC-3 prostate cancer cells.

Cisplatin (μM)	PES (μM)	CI (*n* = 3)	SEM	Fa (*n* = 3)	SEM
20	15	0.665	0.114	0.855	0.026
20	10	0.627	0.164	0.814	0.013
20	5	0.549	0.072	0.758	0.027
10	15	0.689	0.073	0.829	0.027
10	10	0.767	0.065	0.729	0.009
10	5	0.799	0.126	0.583	0.032
5	15	0.927	0.097	0.755	0.036
5	10	1.164	0.064	0.577	0.020
5	5	1.232	0.190	0.262	0.116
2.5	15	0.940	0.080	0.743	0.030
2.5	10	1.333	0.275	0.531	0.030
2.5	5	1.668	0.262	0.268	0.092
1	15	1.058	0.118	0.705	0.027
1	10	1.401	0.234	0.484	0.011
1	5	1.721	0.108	0.224	0.011

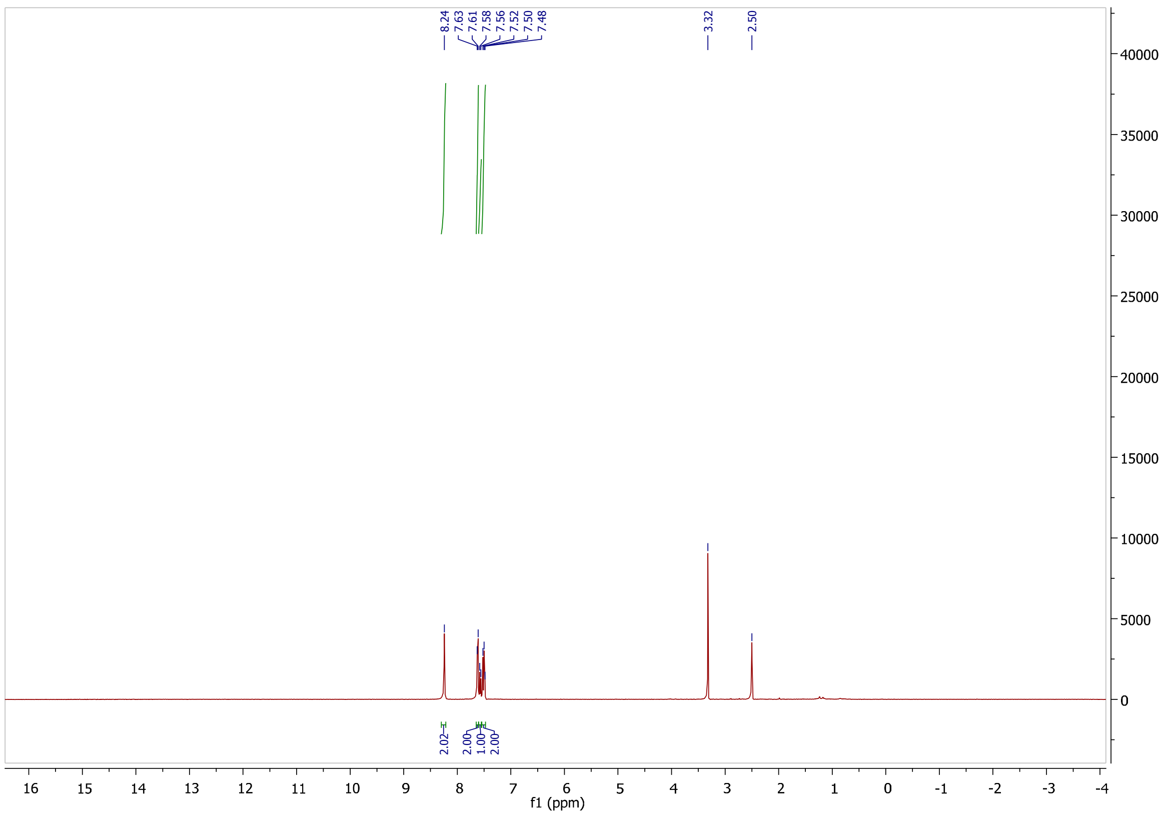

Figure S1 ¹H-NMR spectrum of pifithrin-μ in DMSO-*d*₆.

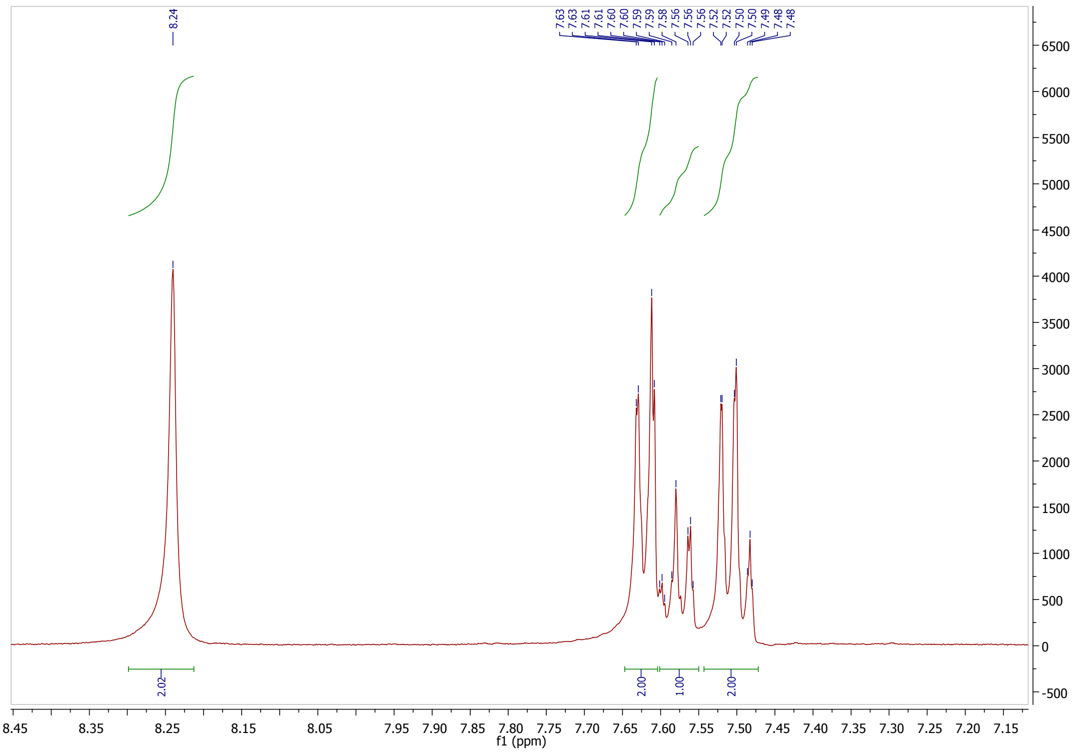

Figure S2. ¹H-NMR spectrum of pifithrin-μ in DMSO-*d*₆.

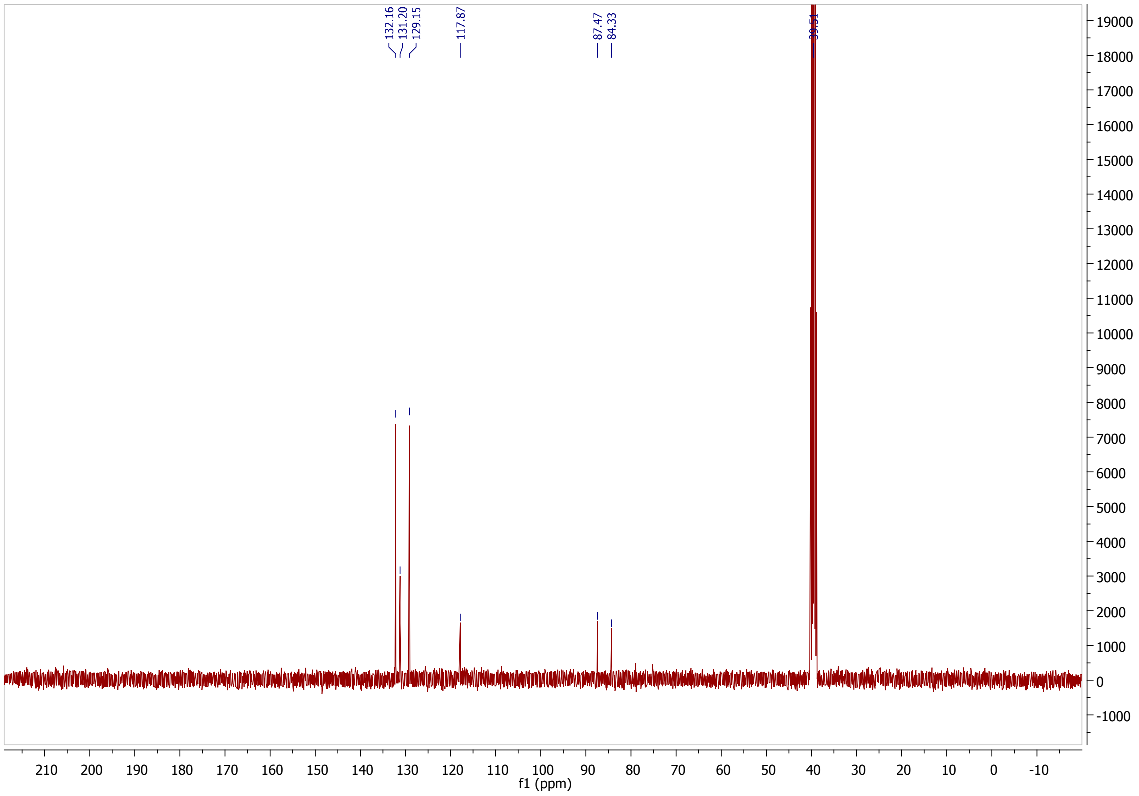

Figure S3. ^{13}C-NMR spectrum of pifithrin-μ in DMSO-*d*6.

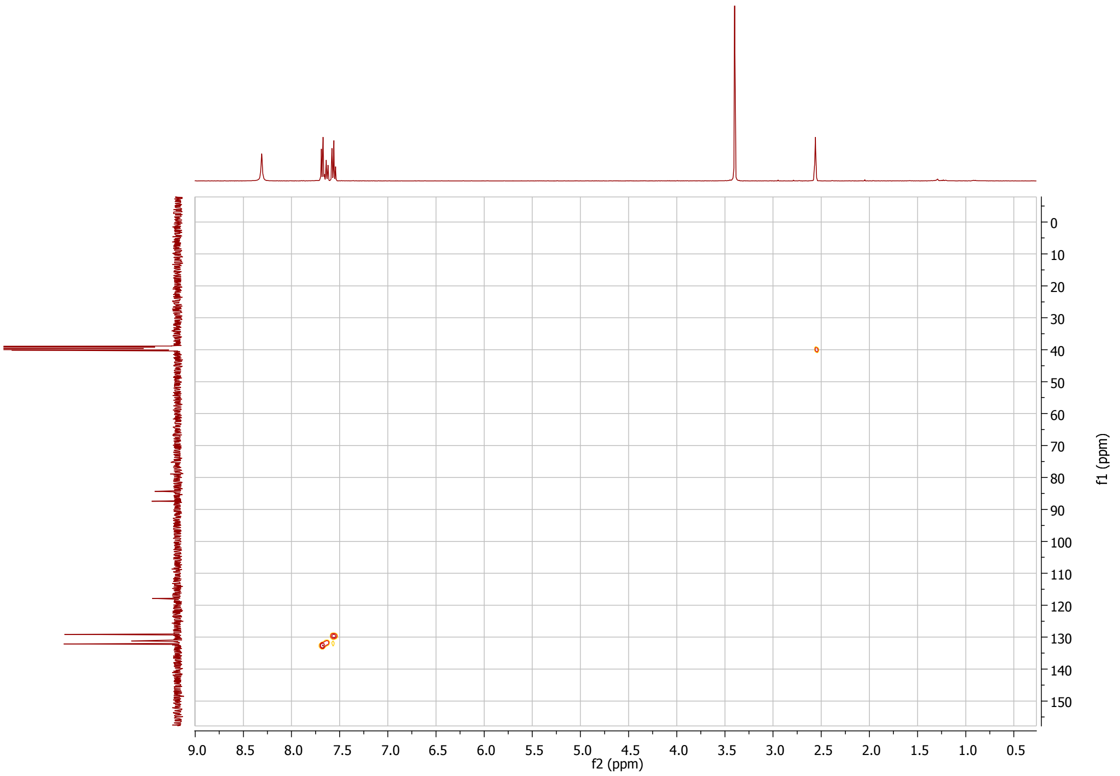

Figure S4. HSQC spectrum of pifithrin-μ in DMSO-*d*6.

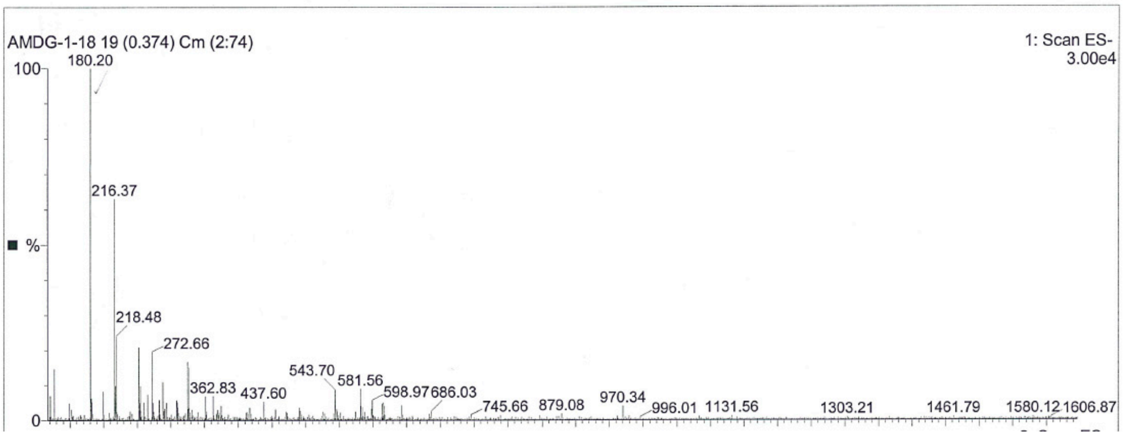

Figure S5. ESI mass spectrum of pifithrin-μ.

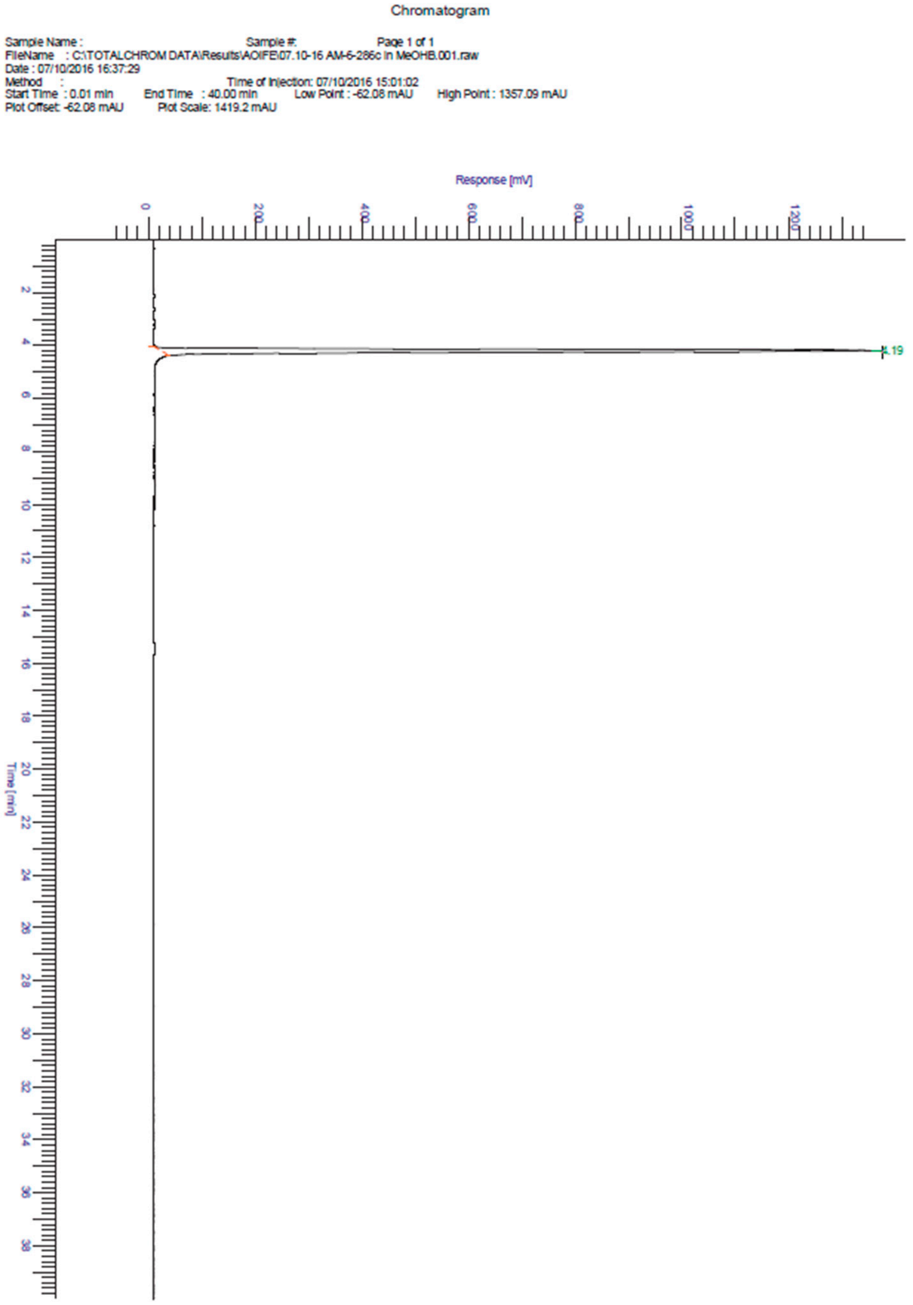

Figure S6. HPLC chromatogram of pifithrin-μ.

Page 1 of 1

Software Version	: 6.3.1.0504		Date	: 07/10/2016 16:37:27
Operator	: manager		Sample Name	:
Sample Number	:		Study	:
AutoSampler	: SER200		Rack/Vial	: 0/4
Instrument Name	: PerkinElmer LC		Channel	: B
Instrument Serial #	: None		A/D mV Range	: 1000
Delay Time	: 0.00 min		End Time	: 40.00 min
Sampling Rate	: 2.2727 pts/s			
Sample Volume	: 1.000000 ul		Area Reject	: 0.000000
Sample Amount	: 1.0000		Dilution Factor	: 1.00
Data Acquisition Time	: 07/10/2016 15:01:02		Cycle	: 1

Raw Data File : C:\TOTALCHROM DATA\Results\AOIFE\07.10-16 AM-6-286c in MeOHB.001.raw
Result File : C:\TOTALCHROM DATA\Results\AOIFE\07.10-16 AM-6-286c in MeOHB.001.rst [Editing in Progress]
Inst Method : C:\TOTALCHROM DATA\Methods\60-40_ACN-Water_1ml_40min from C:\TOTALCHROM DATA\Results\AOIFE\07.10-16 AM-6-286c in MeOHB.001.raw
Proc Method : C:\TOTALCHROM DATA\Methods\60-40_ACN-Water_1ml_40min from C:\TOTALCHROM DATA\Results\AOIFE\07.10-16 AM-6-286c in MeOHB.001.rst [Editing in Progress]
Calib Method : C:\TOTALCHROM DATA\Methods\60-40_ACN-Water_1ml_40min from C:\TOTALCHROM DATA\Results\AOIFE\07.10-16 AM-6-286c in MeOHB.001.rst [Editing in Progress]
Report Format File: C:\PenExe\TcWS\Ver6.3.1\Config\User\manager\Default.rpt
Sequence File : C:\TOTALCHROM DATA\Sequences\ff-.ad.B10%.1ml.-.-20161006-144053.seq

DEFAULT REPORT

Peak #	Component Name	Time [min]	Area [uV*sec]	Height [uV]	Area [%]	Norm. Area [%]	Cal. Range	Volt Range	BL	Raw Amount	Adjusted Amount
1		4.185	10584198.01	1.33e+06	100.00	100.00			BB	10.5842	10.5842
			10584198.01	1.33e+06	100.00	100.00				10.5842	10.5842

Missing Component Report

Component	Expected Retention (Calibration File)

All components were found

Figure S7. HPLC report for pifithrin-μ.
